# The Influence of Spray Cooling Parameters on Workpiece Residual Stress of Turning GH4169

**DOI:** 10.3390/ma17122876

**Published:** 2024-06-12

**Authors:** Xinmin Feng, Jinrong Liu, Jingshu Hu, Zhiwei Liu

**Affiliations:** Key Laboratory of Advanced Manufacturing Intelligent Technology, Ministry of Education, Harbin University of Science and Technology, Harbin 150080, China; fxmin7301@hrbust.edu.cn (X.F.); 2320110084@stu.hrbust.edu.cn (J.L.); 2320110124@stu.hrbust.edu.cn (Z.L.)

**Keywords:** residual stress, parameters spray cooling, GH4169, simulation model

## Abstract

To effectively reduce residual stresses in GH4169 workpieces, thus enhancing fatigue strength and operational lifespan, this study investigates the influence of spray cooling parameters on surface residual stresses during GH4169 turning in spray cooling conditions, utilizing both simulation and experimental approaches. A simulation model of residual stresses was established using finite element analysis when GH4169 was cut in spray cooling. The effects of spray pressure and flow rate on residual tensile stresses were analyzed. The analysis reveals that with increasing spray pressure, residual tensile stresses show a decreasing trend, gradually stabilizing. Conversely, with an increasing spray flow rate, residual tensile stresses initially decrease and then increase. The turning experiments of GH4169 were conducted under different spray parameters. After the experiment, the workpiece was sectioned and analyzed for residual stresses using X-ray diffraction instrumentation. The value residual stress measured closely matched those of simulation, with a relative error within 6%, validating the accuracy of the simulation model and confirming the appropriateness of parameter settings. These results contribute to the further promotion of spray cooling technology and facilitate the rational selection of spray parameters.

## 1. Introduction

With the rapid development of the aerospace industry, many high-temperature alloys with superior performance are gradually becoming necessary materials for processing parts. Nickel-based high-temperature alloys have good structural stability, strong oxidation and corrosion resistance, and low thermal conductivity due to the rich variety of internal elements in their structure. These properties make it adapt high-temperatures, corrosion, oxidation, and other conditions in aerospace operations [1]. However, nickel-based high-temperature alloys with excellent performance often face high processing temperatures that are not easily diffused, severe tool wear, and high residual stresses in the workpiece during processing, which in turn affect the fatigue life of the parts [2]. Many studies show that residual tensile stress can lead to the formation of tiny cracks on the surface or inside the parts, causing unpredictable deformation and misalignment, thereby reducing the fatigue limit and fatigue life of the workpiece, decreasing part accuracy, and increasing the crack expansion rate. In contrast, residual compressive stress exerts the opposite effect, in appropriate amounts, it can inhibit crack initiation, enhance fatigue resistance, reduce or prevent surface defects such as slip bands and deformation, and improve surface finish and flatness, leading to an improvement in the dimensional and geometric accuracy of components [3]. In real production, reducing residual tensile stress helps to decrease cutting heat, allowing mechanical stresses to play a primary role, enhancing the effect of residual compressive stresses, and improving the material’s fatigue limit and machining accuracy. Consequently, studying the variation in residual stress through simulation and experiments is advantageous for enhancing the service life, accuracy, and excellent surface quality of machined parts [4].

In the study of residual stress, it is found that residual stress is mainly generated by plastic deformation caused by mechanical stress and thermal stress, as well as volume changes caused by phase transformation [5]. During the cutting process, a large amount of cutting heat is generated, leading to an increase in temperature in the cutting zone. This results in thermal softening expansion of the cutting layer metal and phase transformation of the metallographic structure, leading to the generation of residual stress. Therefore, the variation in residual stress is usually closely related to cutting heat and cutting forces during the cutting process. Researchers at home and abroad have explored the impact of changes in cutting forces and cutting heat on residual stress by applying cutting fluids to alter the cutting environment. In recent years, the concept of green cutting has gradually gained popularity. Green cutting not only achieves lubrication and cooling effects but also reduces harm to the environment and workers. High-pressure cooling, spray cooling, and cold air cooling are among the most common methods. Researchers have found that these cooling methods promote the reduction of residual tensile stress on the workpiece surface.

Among these cooling methods, high-pressure cooling is the most studied. Li et al. [6] conducted experiments on cutting GH4169 (similar to Inconel 718) with PCBN tools under high-pressure cooling conditions. The study found that the influence of high-pressure cooling fluid on the surface roughness of the machined workpiece was relatively small, which was more helpful in reducing residual surface stress. Another study by Li et al. [7] indicated that under high-pressure cooling conditions, the maximum residual compressive stress strain depth in the cutting depth direction and the maximum residual tensile and compressive stress on the machined surface are both smaller than those in dry cutting. Oliveira et al. [8] found in the research on milling Inconel 718 that residual stress is closely related to variables such as tool wear, cutting force, and cutting heat. In the study of turning GH4169 under high-pressure cooling conditions, Wu et al. [9] utilized Deform-3D to construct a thermal–mechanical coupled finite element model for high-pressure cooling machining of GH4169, analyzing the turning temperature and surface residual stress. The results indicate that with an increase in cooling pressure, the residual tensile stress on the machined surface decreases.

There are some studies on air cooling and MQL (minimal quantity lubrication). Ji et al. [10] found that a larger maximum residual compressive stress was obtained at a low flow rate, small cutting depth, and appropriate gas–oil mixture ratio during the cutting of AISI 413 under air cooling + MQL conditions. Khaliq et al. [11] investigated the lubrication and cooling effects of a tungsten carbide end milling cutter for the micro-milling of Ti-6Al-4V under the condition of micro-lubrication at a low temperature. It was found that there was no significant difference in residual stress values between dry cutting and low-temperature micro-lubrication during micro-milling due to the low cutting temperature. Muhammad et al. [12] utilized dry ice jet cooling during the milling of AISI52100 tool steel and compared the cutting performance with that under micro-lubrication. The results indicated that the residual compressive stress values with dry ice cooling increased by 3% and 8% at speeds of 75 m/min and 300 m/min, respectively, compared to MQL.

There are also some related studies on spray cooling. Yao et al. [13] found that increasing the air pressure could reduce the diameter of the spray droplets, enhancing their penetration ability and enabling easier reduction in the tool’s temperature. Under the condition of spray cooling, Ramanuj et al. [14] employed coated carbide cutting inserts to machine AISI D2 steel. It was discovered that the spray cooling process effectively absorbed a substantial amount of cutting heat during the evaporation phase, resulting in a reduction in cutting temperature. Consequently, this led to a decrease in tool wear and the attainment of superior surface quality. Ukamanal et al. [15] conducted an orthogonal experimental study on the influence of spray cooling process parameters on the machining performance of AISI 316 steel. The study revealed that compared to dry turning, spray cooling resulted in better surface finish and reduced tool wear. Furthermore, the most effective spray pressure was identified.

In the study on the influence of GH4169 material properties, Li et al. [16] analyzed the effects of cutting temperature and load under the mechanical–thermal coupling on the residual surface stress, the plastic deformation degree, and the high-temperature fatigue performance of GH4169 specimens. Yu et al. [17] studied the microstructural evolution during high-speed cutting of the high-temperature alloy GH4169 and found that the dynamic recrystallization (DRX) grain size and volume fraction increased with the rise in cutting temperature, which was attributed to the promotion of grain growth by the elevated temperature.

In the research process of residual stress, due to the numerous factors influencing residual stress and the cumbersome measurement process, researchers often employ various methods such as establishing predictive models, finite element simulations, and so on to investigate changes in residual stress.

Zhou et al. [18] employed a stress relaxation analysis algorithm combined with a finite element model to acquire residual stress distribution data. Additionally, an enhanced BP neural network agent model was utilized for the quick estimation of residual stress. The effectiveness of the hybrid model was ultimately confirmed through cutting experiments conducted on H13 steel. In order to investigate the distribution of surface residual stresses along the feed direction during turning, Weng et al. [19] employed a three-dimensional numerical model based on the Coupled Eulerian–Lagrangian (CEL) method to accurately predict the evolution of residual stresses during multiple consecutive cuts in turning operations. The effectiveness and accuracy of the proposed model were validated through a strong agreement between simulation results and experimental measurements. Ullah et al. [20] proposed a numerical and experimental approach to comprehensively predict the residual stresses in milled sections of Ti-6Al-4V alloy. Upon establishing the model, excellent correlation was achieved between simulation and experimental results for given milling conditions. Finally, the impact of the white layer on residual stress distribution was investigated. This analytical method provides a thorough understanding of residual stress distribution within milled components.

Li et al. [21] utilized multiple regression analysis to develop a residual stress prediction model for rough turning Ti-6Al-4V. This model takes into account not only cutting parameters but also cutting force and cutting temperature. Through model analysis, it is evident that the friction coefficient and tool edge radius influence the thickness of the residual stress layer. While cutting speed has minimal impact on the thickness of the residual stress layer, an increase in cutting speed can induce a shift from residual stress to tensile stress, eventually leading to residual stress approaching zero at a specific depth.

Although residual stresses can be obtained through predictive modeling and experimentation, the process often demands significant computational time and material wastage. Utilizing finite element simulation not only allows for precise analysis of the workpiece material’s plastic deformation but also enables visualization of the material’s evolution during the process, leading to more accurate simulation results. For instance, Alok et al. [22] used a finite element model to simulate the effect of cutting speed and cutting depth on residual stresses when turning Ti-6Al-4V. The accuracy of the model was verified by comparing it with experimental values. Luo et al. [23] employed the Third Wave AdvantEdge finite element analysis software to conduct simulation experiments on turning 7075-T651 aluminum alloy. The aim was to investigate the influence of cutting parameters, such as cutting depth and feed rate, on cutting force, residual stress, and cutting temperature. The results obtained from the simulations align well with the experimental values. Min et al. [24] summarized the methods for calculating residual stress from different contact conditions based on temperature, elastic stress, and plastic stress, and analyzed the advantages and disadvantages of analytical methods and finite element methods.

Based on the research conducted by the aforementioned scholars, it is understood that residual stress is closely associated with various factors including turning parameters, tool specifications, workpiece materials, and cooling conditions. The cooling environment directly influences the thermal effects during the cutting process, consequently mitigating residual tensile stresses, fatigue life, cracks, and precision-related phenomena. However, the influence of spray cooling parameters on residual stress changes in GH4169 has received limited attention from researchers. Hence, this paper employs finite element simulation to investigate the variation patterns of residual stress in GH4169 workpieces under different spray parameters (such as pressure and flow rate). This research aims to provide insights for subsequent studies on workpiece surface integrity.

## 2. Establishment of Finite Element Model

### 2.1. The Establishment of Material Constitutive Model and Friction Model

The material model is an important component of finite element simulation, and whether the simulation results are accurate or not depends largely on the accuracy of the constitutive model; therefore, the Power Law material model was selected [25] to carry out the simulation, and the established material constitutive equations are shown in Equation (1):

(1) $$\sigma (\varepsilon^{p},\varepsilon ,T)=g(\varepsilon^{p})\Gamma (\varepsilon )\theta (T)$$

where $g(\varepsilon^{p})$ is the strain intensification function; $\Gamma \left(\varepsilon \right)$ is the stress rate effect coefficient; θ(T) denotes thermal softening function.

In the Third Wave AdvantEdge (7.1.002) simulation process, strain hardening, thermal softening, and strain will gradually lead to failure as the cutting process progresses. To assess this, failure criteria for the three functions need to be applied, as shown in Equations (2)–(4), which represent the failure equations for strain hardening, thermal softening, and strain functions, respectively.

(2) $$g\left(\varepsilon^{p}\right)=\sigma_{0}{\left(1+\varepsilon^{p}/\varepsilon_{0}^{p}\right)}^{1/n}$$

(3) $$\theta \left(T\right)=C_{0}+C_{1}T+C_{2}T^{2}+C_{3}T^{3}+C_{4}T^{4}+C_{5}T^{5}$$

(4) Γε=1+ε/ε01m1

where $\sigma_{0}$ is the value of yield residual stress when uncut; $\varepsilon_{0}^{p}$ is the plastic strain rate reference value; *n* is the coefficient of plastic deformation; $C_{0}$~$C_{5}$ are the polynomial coefficients; and *m*_1_ is the coefficient of strain rate at low strains.

**Figure 1:**
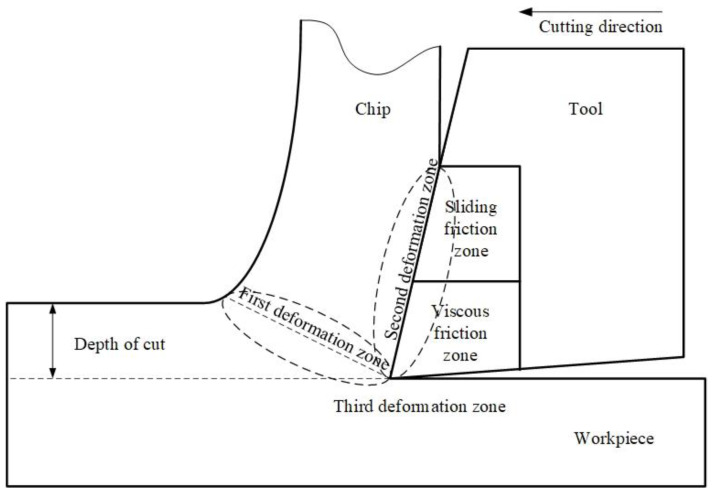
Coulomb friction model.

The primary factor influencing the formation of residual tensile stress is the cutting heat, and a large amount of frictional heat is generated between the tool–chip and tool–workpiece during cutting. Therefore, a reasonable friction model for the tool–chip contact area is crucial for obtaining accurate residual stress changes. The tool–chip interface in the Third Wave AdvantEdge simulation software adopts a Coulomb friction model, where the sum of the two friction lengths in the adhesive and sliding regions is the total length of the tool–chip contact, depicted in Figure 1 [26].

The Coulomb friction model is represented by Equations (5) and (6):

(5) $$\tau_{f}\left(x\right)=\sigma_{p}$$

(6) $$\tau_{f}\left(x\right)=\mu \sigma_{n}\left(x\right)$$

where $\sigma_{n}$ is the normal stress; $\sigma_{p}$ is the flow stress of the material; μ is the coefficient of friction.

The friction coefficient needs to be entered in the parameter settings of the simulation software. The friction coefficient is mainly related to the tool rake angle *θ* and the cutting force, as shown in Equation (7):

(7) $$\mu =C\times \frac{F_{t}\sin\theta +F_{c}\tan\theta }{F_{c}\cos\theta +F_{t}\tan\theta }$$

where μ is the friction coefficient; $F_{t}$ is the axial cutting force; $F_{c}$ is the radial cutting force and C is a constant. According to the related paper [27], the friction coefficient of spray cooling condition is 0.3.

In the research on orthogonal cutting force modeling, based on the phenomenon of high temperature and high strain during the cutting of nickel-based high-temperature alloy materials, the Oxley model and Power Law model are usually used in combination when cutting force modeling is carried out. When the shear angle is known, the tool–chip friction force, plow force, and cutting force models can be obtained from the combined Oxley and Power Law model and further calculate the tool–workpiece frictional heat [28]. However, in thermal simulation modeling of cutting heat, apart from the heat generated between the tool and workpiece due to friction, there is also heat generated by shear bands and temperature changes when applying coolant. When applying coolant, $\theta_{cool}(X,Z)$ can be treated as a fixed heat source model causing temperature variations for prediction, as shown in Equations (8) and (9):

(8) $$\theta_{cool}(X,Z)=\frac{q_{cool}}{2\pi K_{t}}\int_{0}^{1}\int_{\frac{-w}{2}}^{\frac{w}{2}}(\frac{1}{R_{i}}+\frac{1}{R_{i}^{'}})dydx$$

(9) $$q_{cool}=\bar{h}(T-T_{0})$$

where $q_{cool}$ is the intensity of heat loss; $\bar{h}$ is the total heat transfer coefficient; $T_{0}$ is the ambient temperature.

The final temperature of the workpiece results from the sum of the two heat sources and the cooling model. Based on the analysis above, it is evident that obtaining the convective heat transfer coefficients when applying cutting fluid in the model is essential. Residual stresses are caused by plastic deformation induced by changes in cutting forces and cutting heat. The application of spray cooling has a positive influence on the workpiece, contributing to achieving excellent surface quality and an improved cutting environment.

### 2.2. Mesh Delineation and Simulation Parameter Settings

#### 2.2.1. Parameters Setting

**Figure 2:**
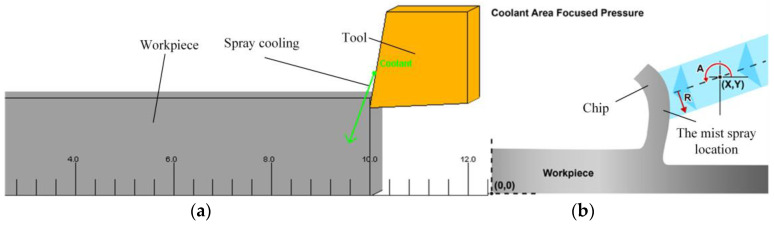
The finite element turning simulation model. (**a**) Turning model. (**b**) Spray cooling.

The finite element turning simulation model is shown in Figure 2a, which can be viewed as the addition of countless cross sections when the workpiece is rotating. So, the workpiece can be viewed as a simple rectangle when it is moving in the direction of the tool feed, and the 2D planar model can be transformed from the 3D machining model. Figure 2b is the schematic diagram of the position of the nozzle and the direction of the spray when the coolant is working.

Some main parameters were set in the 2D cutting module of the Third Wave AdvantEdge pre-processing module.

Due to the limitation of Third Wave AdvantEdge software in only being able to set the coolant flow velocity to change the cooling conditions, the representation of spray cooling can be achieved by changing the convective heat transfer coefficient and the flow rate of the cutting fluid. In the calculation process, the diameter of the nozzle can be considered as the cross-sectional area through which the cutting fluid passes, and the flow velocity can be obtained for different pressures and flow rates through the conversion of flow velocity using Equations (10)–(12).

(10) $$Q=C_{d}\cdot n\cdot \frac{\pi d^{2}}{4}\cdot \sqrt{\frac{2P}{\rho }}$$

(11) $$V=\frac{Q}{S_{A}}$$

(12) $$S_{A}=\frac{\pi d^{2}}{4}$$

where *V* is the fluid flow velocity; *C_d_* is the working efficiency of the nozzle, where *C_d_* is taken as 1; *n* is the number of nozzles; *d* is the diameter of the nozzle; ρ is the density of cutting fluid; *P* is the pressure; *Q* is the flow rate at the nozzle; and *S_A_* is the area of the aperture at the nozzle.

**Figure 3:**
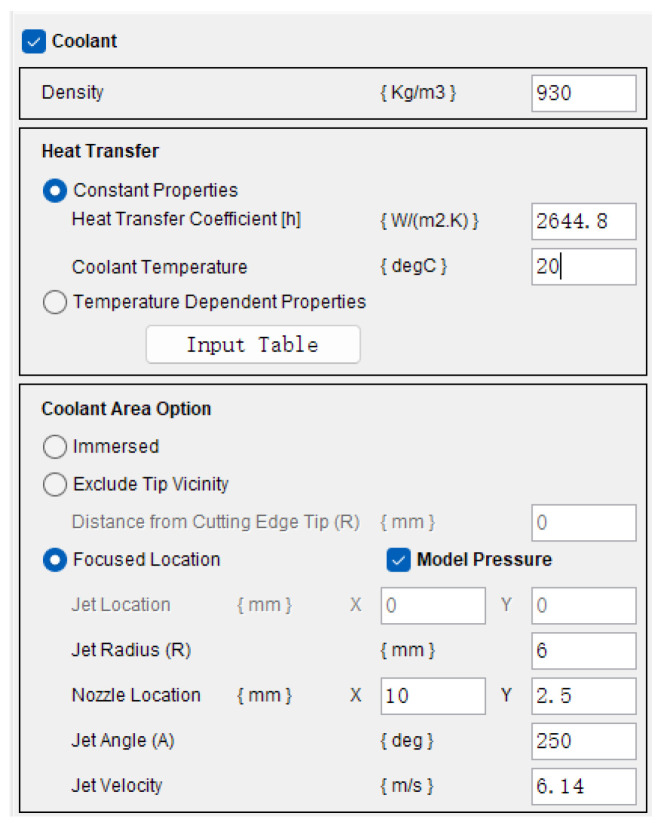
Parameters setting of cooling medium.

At a flow rate of 2.5 L/h, the cooling parameters are set as shown in Figure 3, with the nozzle location at X10, Y2.5, a jet angle of 250°, a convective heat transfer coefficient of 2644.8, and a jet velocity of 6.14 m/s. This configuration allows for a more accurate simulation of the application of spray cooling in turning environments. Secondly, the cutting speed is 100 m/min, the feed rate is 0.2 mm/r, the depth of cut is 0.25 mm, and the depth of mesh refinement for residual stress analysis is 0.4 mm. Process option parameters are selected as general. In the “Define Friction Coefficient” section, a friction coefficient of 0.3 is selected. Furthermore, the tool is adequately constrained in the X and Y directions and the number of cuts for residual stress is set to 2 to consider the influence of the previous cut on the next one. Finally residual stress simulation is performed under the assumption of solely considering thermodynamic effects and neglecting material defects.

The convective heat transfer coefficient is related to the density and viscosity coefficient of the fluid. Through the previous study [29], ANSYS (2020.R2) fluid simulation was used to obtain the convective heat transfer coefficient values of different pressures and flow rates during the spray cooling.

#### 2.2.2. Mesh

**Figure 4:**
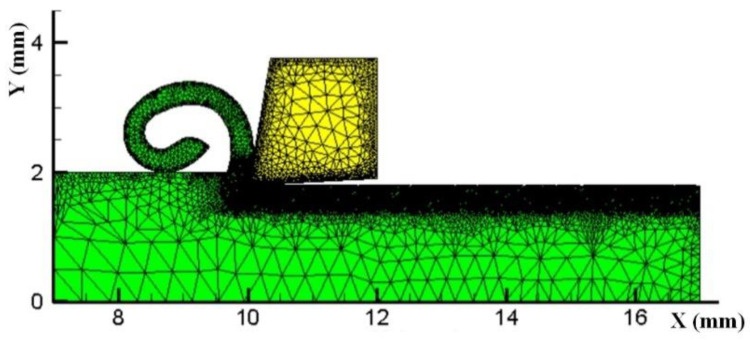
Mesh of tool and workpiece.

In the Third Wave AdvantEdge software, the Lagrangian method with adaptive and continuous mesh partitioning is employed to accurately describe the motion of structural boundaries. The mesh coarsening factor is used to control the rate at which the mesh coarsens to its maximum size, thereby affecting the degree of coarsening after element deformation. Conversely, the refinement factor determines the rate at which the mesh refines to its minimum size, thereby influencing the degree of mesh refinement. In this study, the mesh coarsening and refinement factors were set to 6 and 2, respectively. Subsequently, the mesh sizes for the tool and workpiece regions were defined, with a minimum mesh element size of 0.02 mm and a maximum element size of 0.1 mm. The range of values for the mesh partitioning level parameter G is from 0.1 to 1, where its magnitude determines the rate of transition from coarse to fine mesh near the cutting-edge region. After considering a comprehensive evaluation of simulation time and accuracy, a value of G was 0.4. The mesh partitioning results are illustrated in Figure 4.

## 3. Finite Element Simulation Results Analysis

**Figure 5:**
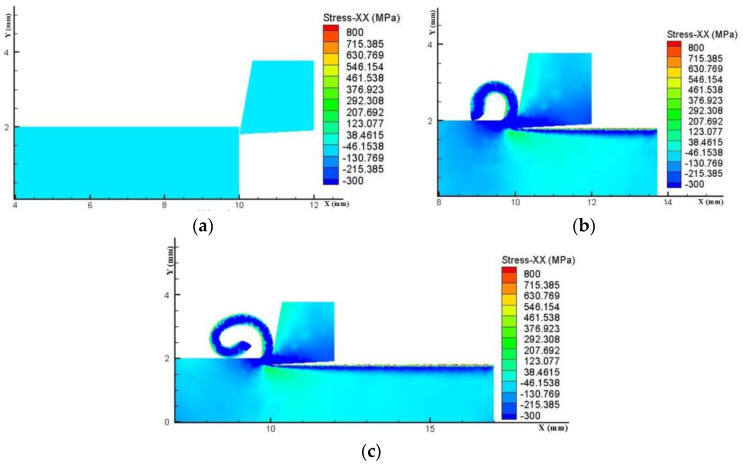
Variation of residual stress in turning. (**a**) Initial stage of turning machining. (**b**) Intermediate stage of machining. (**c**) Residual stress diagram after cooling and unloading.

The residual stress on the workpiece surface after the turning process is the result of four stages: turning, unloading, constraint transformation, and cooling. Simulation investigates the effects of pressure and flow rate of spray cooling using a single-factor experimental design, with the accuracy of this model validated through experimentation. The process of residual stress change in turning machining is shown in Figure 5. The software simplifies the cutting section of the workpiece into a rectangle, where the horizontal axes represent the position of the tool in the cutting direction and the vertical axes denote the cutting depth. The color scale indicates the magnitude of residual stresses, with positive values representing residual tensile stresses and negative values indicating compressive stresses. On the surface of the machined workpiece, a transition from red-colored residual tensile stresses at the surface gradually decreasing towards the interior of the workpiece and becoming blue-colored residual compressive stresses can be observed, eventually reaching a pale blue color indicating a stress-free state.

### 3.1. Effect of Spray Cooling Pressure on Residual Stresses

**Figure 6:**
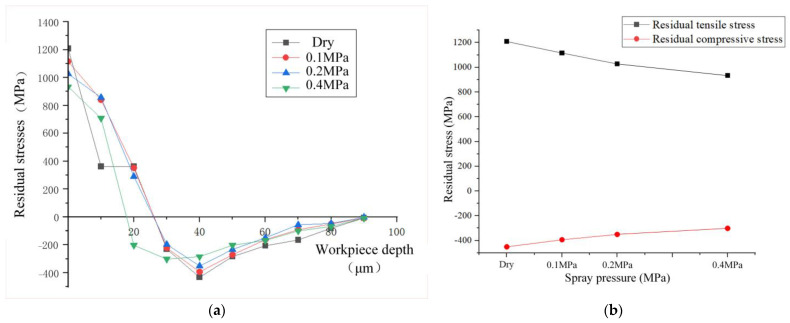
Residual stress at different pressures. (**a**) Residual stress variation trend chart under different spray pressure. (**b**) Maximum residual stress variation chart under different spray pressure.

When the cutting speed is 100 m/min, feed rate is 0.2 mm/r, and cutting depth is 0.25 mm, the variation trend of residual stress in the cutting depth direction of the workpiece with depth by two-dimensional simulation is shown in Figure 6. From the figure, it can be observed that the workpiece surface exhibits residual tensile stress. In dry cutting, the residual tensile stress reaches 1208.5 MPa. During spray cooling conditions, the residual tensile stress values at spray pressures of 0.1 MPa and 0.2 MPa are 1115.51 MPa and 1027.05 MPa, respectively. The minimum residual tensile stress value of 933.93 MPa is achieved at a spray pressure of 0.4 MPa, resulting in a relative reduction of 22.72% compared to dry cutting. The residual tensile stress decreases as the spray pressure increases from 0.1 MPa to 0.4 MPa. Compared to dry cutting, spray cooling offers significant advantages in cooling and protecting the workpiece surface. Initially increasing the spray cooling pressure allows the cutting fluid to enter the tool–chip contact area, forming an oil film on the surface that effectively reduces the friction coefficient between the tool and chip, thereby decreasing the friction and cutting heat. Additionally, increasing the pressure can enhance the convective heat transfer coefficient. Since air is the carrier of the cooling pressure, the cooling efficiency primarily depends on the specific heat of the gas. Coolants with higher specific heat values can absorb more heat from the workpiece and cutting tool. Air has a relatively high specific heat, so increasing pressure can expedite cooling, leading to a reduction in the cutting temperature of the machined surface. These factors contribute to a gradual decrease in residual tensile stress with increasing spray pressure between 0.1 MPa and 0.4 MPa.

**Figure 7:**
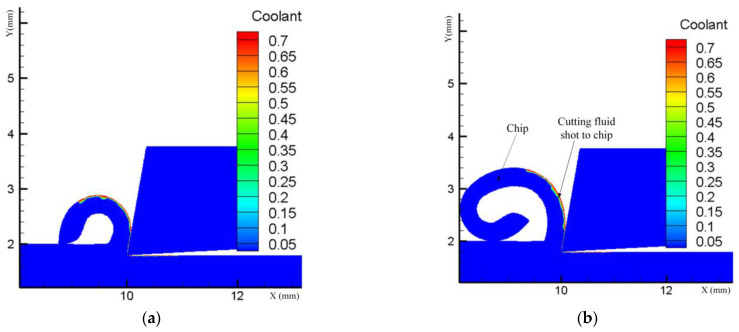
Volume fraction of cutting fluid on chips at different stages of cutting. (**a**) Diagram of the initial effect of pressure on chip formation. (**b**) Diagram of the effect of pressure on the stabilization of chip intermediate formation.

From Figure 6, it is evident that the change in residual compressive stress between pressures of 0.1 MPa and 0.4 MPa shows a decreasing trend as pressure increases. This phenomenon can be elucidated as follows: Firstly, during the turning process, the poor thermal conductivity of GH4169 results in significant heat generation at the tool nose, leading to thermal softening on the workpiece surface and subsequently reducing the cutting force. Secondly, the orientation and angle of the spray jet directed towards the rake face facilitate the fracture of serrated chips during turning, thereby reducing cutting forces and mechanical stresses. Consequently, an increase in pressure results in a decrease in residual compressive stresses. The fraction of the cutting fluid on the chip at various moments during spray cooling is depicted in Figure 7. The horizontal axis represents the position of the tool in the cutting direction, while the vertical axis corresponds to the depth of cut in the axial direction. When observing changes in cutting depth, it is noted that the thermal effect diminishes under a pressure of 0.4 MPa. The depth at which the transition from residual tensile stress to residual compressive stress occurs is advanced to approximately 20 μm, with the maximum residual compressive stress advancing by 10 μm compared to pressures of 0.2 MPa and 0.1 MPa, reaching around 30 μm.

### 3.2. Effect of Flow Rate of Spray Cooling on Residual Stresses

**Figure 8:**
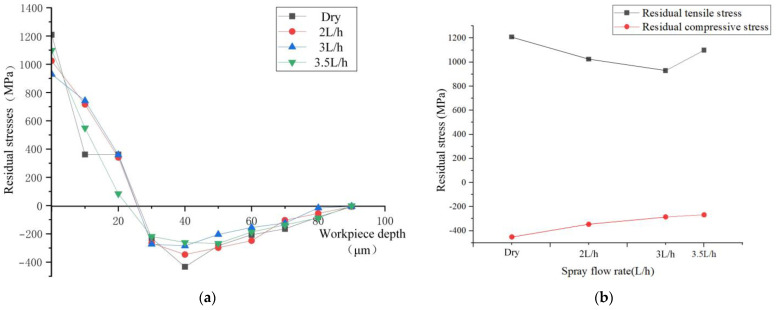
Residual stress at different flow rates. (**a**) Residual stress variation trend chart under different flow rates. (**b**) Maximum residual stress variation chart under different flow rates.

The variation in residual stress with the flow rate of spray cooling is depicted in Figure 8. As the spray cooling flow rate increases within the range of 2 L/h to 3.5 L/h, the residual tensile stress value demonstrates a trend of initially decreasing and then increasing. The maximum residual tensile stress values at spray flow rates of 2 L/h, 3 L/h, and 3.5 L/h are 1023.95 MPa, 928.98 MPa, and 1099.15 MPa, respectively. At a flow rate of 3 L/h, the minimum residual tensile stress is achieved, with a relative reduction of 23.13% compared to dry cutting.

Unlike increasing the air pressure, increasing the flow rate not only enhances the cooling effect but also impacts the tool wear rate. An increase in the flow rate enhances the lubrication effect at the cutting point, consequently reducing the tool wear rate. Simultaneously, it diminishes the heat generated during cutting due to wear, thereby reducing the residual stresses on the workpiece’s cutting surface. Moreover, decreasing tool wear hampers the escalation of cutting forces and enhances the quality of the machined surface, ensuring that residual stresses remain minimal and evenly distributed.

**Figure 9:**
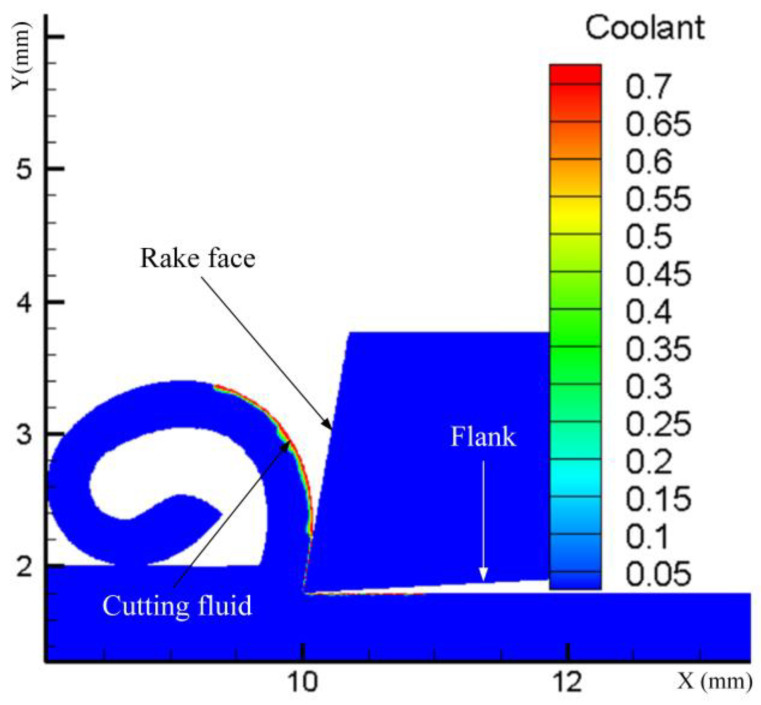
Diagram of spray into the tool–chip, tool–workpiece contact area.

Through simulation, it has been observed that an increase in flow rate leads to a decreasing trend in residual tensile stress within the range of 2 L/h to 3 L/h. As illustrated in Figure 9, during the cutting process, the second deformation zone generates a region with elevated temperature. The cutting fluid sprayed on top of this region rapidly vaporizes and volatilizes under the high temperature, thereby hindering its lubricating function. By augmenting the spray flow rate, a substantial amount of cutting fluid flows into the tool–workpiece contact area, carrying away the heat generated during cutting. Furthermore, excess cutting fluid infiltrates the tool–chip contact area to fully exploit its lubricating effect. The cutting fluid serves to enhance the frictional conditions, reducing both the coefficient of friction and frictional force. The reduction in frictional force subsequently helps to constrain the cutting heat, thereby influencing the tool wear rate. Additionally, by mitigating the cutting heat, it also impacts the tool wear rate.

Residual stress is the result of mechanical–thermal coupling, and the simulation results from spray pressure and flow rate indicate that spray cooling can enhance cutting conditions, optimizing residual stress. Compared to dry turning, at an optimal spray pressure of 0.4 MPa, the residual tensile stress decreased by 22.72%, and the residual compressive stress increased by 26.54%. When the optimal spray flow rate was 3 L/h, the residual tensile stress decreased by 23.13%, while the residual compressive stress increased by 30.84%.

The spray cooling process involves the mixture of gas and cutting fluid to form a mist for lubrication and cooling purposes. As the proportion of cutting fluid continuously increases and reaches an optimal state with the gas, there is a decreasing trend in residual tensile stress within the range of 2 L/h to 3 L/h. Increasing the flow rate of cutting fluid disrupts the effective lubrication in the cutting zone, leading to a decrease in lubrication effectiveness. Therefore, a significant increase in residual tensile stress is observed when the flow rate increases from 3 L/h to 3.5 L/h. And the overcoming of frictional forces between the tool and chips is one of the sources of cutting forces. Changes in frictional forces result in a decrease in cutting forces, leading to a reduction in residual compressive stresses in the workpiece due to the decrease in mechanical stress and thermal stress. As shown in Figure 8, the maximum residual compressive stress values are all less than those during dry cutting, and they decrease with an increase in flow rate.

By comparing the residual stress variation curves at different spray flow rates, the maximum residual compressive stress at a cutting fluid flow rate of 3.5 L/h is 267.81 MPa. Compared to 2 L/h and 3 L/h, the position of the maximum residual compressive stress increases, which is located near the workpiece surface at around 50 μm. The main reason for this shift is the decrease in effective lubrication, which leads to an increased influence range of cutting heat, thereby altering the position of the maximum residual compressive stress.

### 3.3. Analysis of the Effect of Spray Cooling

The spray cooling process involves the mixture of liquid and gas, which is then sprayed onto the surface of the turning process through high-pressure atomization. Spray cooling primarily serves to cool and lubricate, as the small oil droplets sprayed from the nozzle reduce the cutting temperature in the cutting zone, on the tool, and on the workpiece, thereby improving the residual stress effect.

From the Leidenfrost phenomenon [30], it can be known that during metal cutting, the high temperatures generated in the cutting zone create a high-pressure insulating vapor layer around it. When cutting fluid is applied, a portion of it evaporates and is lost due to the lower boiling point of the cutting fluid. The cutting fluid entering the cutting zone is also impeded in heat transfer by the vapor layer, significantly reducing the effectiveness of the cutting fluid.

**Figure 10:**
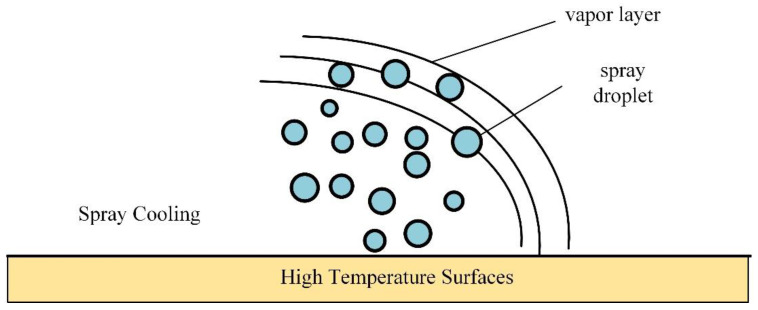
Leidenfrost phenomenon for spray cooling.

Spray cooling, supported by gas pressure, can break through the constraints of the vapor layer. As shown in Figure 10, with enhanced penetration capability, the vapor layer can be penetrated by the lubricant, thereby increasing the convective heat transfer coefficient and heat transfer on the cutting surface. In addition to improving lubrication, spray cooling also enhances the friction coefficient in the tool–chip contact area. The tool–chip contact area is not completely fitted together but can be seen as a capillary phenomenon with small gaps. By applying pressure, the cutting fluid can infiltrate the tool–chip contact area, providing boundary lubrication. Due to the adsorption properties of the boundary lubrication film, it can adhere to the surface of the tool–chip contact, while the lubricating oil film reduces friction by decreasing the cutting heat. In addition to entering the tool–chip contact area, a portion of the spray will also be sprayed onto the contact area between the flank face and the workpiece to reduce the wear and friction on the flank face. Spray and pressure not only improve lubrication but also promote convective heat transfer, lowering the temperature in the cutting zone.

The generation of residual stresses can be considered as a result of mechanical–thermal coupling, caused by the plastic deformation of mechanical stress and thermal stress. Due to frictional forces and plastic deformation, a significant amount of cutting heat is generated in the secondary deformation zone. The use of spray increases the heat transfer rate and improves lubrication and friction, thereby reducing the cutting heat in the secondary deformation zone of the tool–chip contact. This has a promoting effect on reducing residual tensile stresses and can enhance the fatigue life of the workpiece.

## 4. Turning Experiment of GH4169 during Spray Cooling

### 4.1. Selection of Equipment and Materials for Experiment

The model CA6140 lathe was chosen as the experimental platform, and Sandvik’s CNMG120404-SF1105 carbide inserts were used. The workpiece was a Φ120 × 300 mm GH4169 bar, whose elemental composition and physical properties are shown in Table 1 and Table 2.

**Figure 11:**
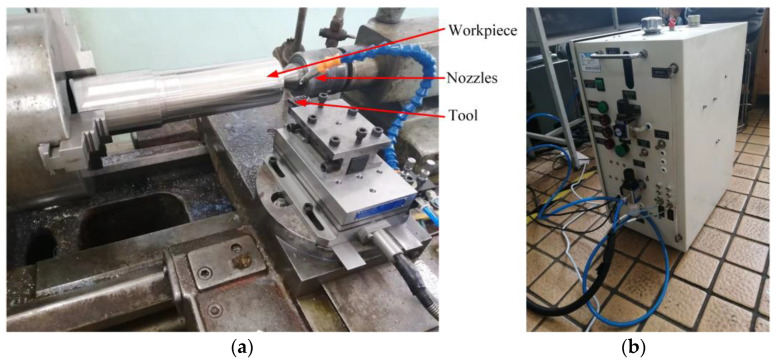
Experimental equipment. (**a**) Processing equipment. (**b**) Spray equipment.

The turning conditions were spray cooling lubrication, realized by the OoW129S composite spray cooling system. The experimental equipment is shown in Figure 11

Based on the optimal cutting parameters determined in previous research, the cutting parameters were set as follows: cutting speed *v* was 100 m/min, feed rate *f* was 0.2 mm/r, and cutting depth *a_p_* was 0.25 mm. The spray pressure was set at 0.1 MPa, 0.2 MPa, and 0.4 MPa, while the spray flow rate was set at 2 L/h, 3 L/h, and 3.5 L/h. Cutting experiments were conducted with fixed cutting parameters while the spray parameters varied. Each cutting was used to machine the workpieces at different positions with the same cutting depth, resulting in workpieces of varying diameters. After completing all cutting experiments, sections of the workpieces with different diameters were cut into slices using wire cutting to create samples. The spray parameters and cutting parameters are shown in Table 3.

### 4.2. GH4169 Residual Stress Measurement

**Figure 12:**
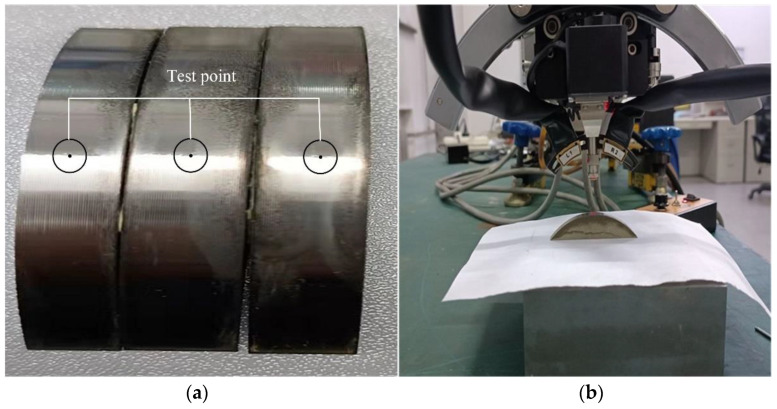
Workpiece Specimen and X-ray stress instrumentation. (**a**) Workpiece specimen. (**b**) X-ray stress instrumentation.

After the turning experiment, the processed bar material was sliced using wire cutting to obtain a workpiece sample, as shown in Figure 12. Residual stress was measured using an IXRD X-ray stress meter. The instrument was capable of achieving an accuracy of up to ±8 MPa, with an X-ray penetration depth ranging from 10 to 20 μm. Due to the directional nature of residual stress, there were variations in residual stress in both the cutting depth and feed directions. However, in order to maintain consistency with the simulation, only residual stress in the cutting depth direction was measured in this paper. In order to reduce measurement errors, the measurement point location was chosen at the midpoint of stable cutting, which effectively avoided the influence of the large cutting force generated by the tool cutting material during the initial cutting and the influence of tool wear at the end of cutting. Additionally, the X-ray diffractometer could only measure surface residual stress. For depth direction residual stress measurement, a stripping process had to be carried out. Firstly, the workpiece was corroded with NH4CL electrolyte in a 6 × 6 mm^2^ area, and then the surface was polished using a polishing machine for 6 s. Residual stress measurements were conducted when the polishing depth reached 10 μm. The sample was placed within the X-ray diffractometer, where X-ray beams were directed onto its surface. The X-rays interacted with the atomic nuclei and electrons within the sample’s crystal structure, resulting in diffraction effects. Utilizing data from diffraction patterns, strain information of the sample’s crystal structure was computed. Based on Bragg’s law and the strain–diffraction angle relationship, residual stresses within the sample could be deduced.

### 4.3. Effect of Spray Cooling Pressure and Flow Rate on Residual Stresses

**Figure 13:**
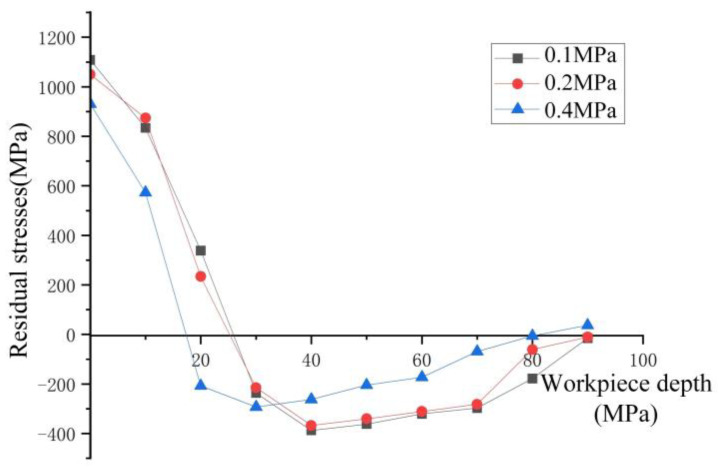
Measured residual stresses at different pressures.

**Figure 14:**
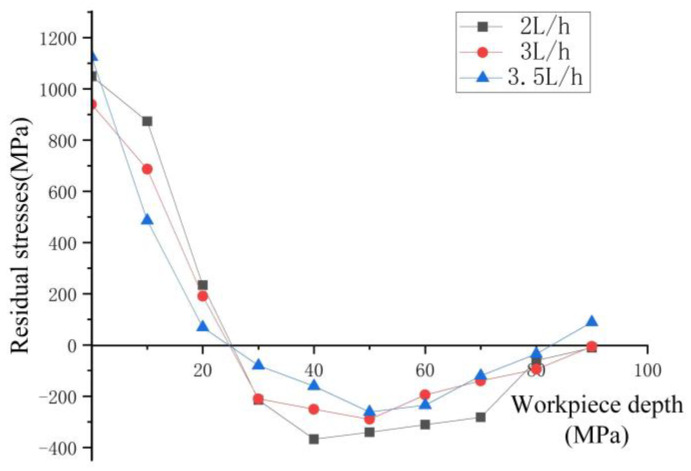
Measured residual stresses at different flow rates.

By measuring the residual stress values, the residual stress trend graphs were obtained, as shown in Figure 13 and Figure 14. The error analysis of the experimental and simulation groups regarding the residual tensile and residual compressive stresses is shown in Table 4.

From the figure, it can be observed that the trend of residual stress values is consistent with the simulated image, showing a spoon-shaped distribution. The experimental results indicate that the residual tensile stress decreases with increasing spray pressure in the range of 0.2–0.4 MPa, while the residual compressive stress increases. Similarly, with increasing spray flow rate in the range of 2–3.5 L/h, the residual tensile stress initially decreases and then increases, while the residual compressive stress increases. The experimental data in the graph show a transition from tensile stress to compressive stress, occurring at around 25 μm, with minor discrepancies compared to the simulated values. Regarding the maximum residual compressive stress and residual stress depth, the experimental curve converges with the simulated curve.

Because residual stress is the result of the mechanical–thermal coupling, spray cooling can help reduce the temperature by dissipating heat, improve chip evacuation, and reduce the impact of heat on the workpiece. Additionally, cutting fluid can decrease friction, thus lowering cutting forces and reducing the extent of plastic deformation, thereby diminishing residual stress.

Through the calculation, it can be seen that the error between the experimental value and the simulation value is kept within 6%. For example, the flow rate of 2 L/h shows the largest error of 5.8% in the compressive stress value. This discrepancy can be attributed to the composition of the nickel-based high-temperature alloy material, which consists of Ni, Cr, Nb, and other major components. The proportions of these components within the workpiece material in actual production may not be identical to the proportions specified, leading to changes in the residual stress values. In the finite element simulation software, the workpiece was set as a completely standard material unaffected by factors such as temperature and oxygen content during the simulation process. This difference in environmental conditions between the simulation and actual cutting processes contributes to the error between the experimental and simulation values. Additionally, the poor thermal conductivity of GH4169 and the close proximity of the samples on the workpiece can result in the cutting heat affecting subsequent cuts during the experimental process. This issue is not present in the simulation, leading to a relatively larger relative error in compressive stresses.

## 5. Conclusions

This paper established a cutting simulation model for the nickel-based superalloy GH4169 during spray cooling, and the influence of flow rate and pressure of spray parameters on residual stress was analyzed. Experimental cutting tests were conducted on GH4169 during spray cooling, and the residual stresses of the specimens were measured. A comparative analysis of the error between the residual stresses obtained from simulation and experimental measurements was performed. The following conclusions were drawn:

(1) The experimentally measured residual stresses were consistent with the trends of the simulated values, with a relative error of less than 6%, validating the accuracy of the simulation model.
(2) Residual stress is the result of the mechanical–thermal coupling, and spray cooling can improve cutting conditions, thereby optimizing residual stress.
(3) When the spray flow rate is 0.2 L/h and the pressure is changed for simulation, it is found that when the spray pressure is 0.4 MPa, the residual stress reaches the optimal value. Compared with dry cutting, the residual tensile and compressive stress are relatively reduced by 22.72% and 26.54%, respectively.
(4) When the spray pressure is 0.2 MPa and the flow rate is changed for simulation, it is found that when the spray flow rate is 3 L/h, the residual stress reaches the optimal value. Compared to dry cutting, residual tensile stress compressive stress was relatively reduced by 23.13% and 30.84%, respectively.
(5) The residual tensile stress on the sample surface decreased with an increase in spray pressure, and the minimum residual tensile stress was obtained when the optimal parameters for spray pressure were 0.4 MPa. Specifically, at this point, the maximum residual compressive stress distance from the workpiece surface decreased, reaching around 30 μm, but the overall trend of residual stresses did not change.
(6) The residual tensile stress on the sample surface initially decreased and then increased with an increase in spray flow rate. The residual tensile stress decreased when the flow rate was between 2 L/h and 3 L/h, and showed an upward trend from 3 L/h to 3.5 L/h, making 3 L/h the optimal spray flow rate. When the spray flow rate reached 3.5 L/h, the maximum residual compressive stress distance from the workpiece surface increased due to the influence of thermal stresses, occurring at around 50 μm, but the overall depth of residual stress change was not affected.

## Figures and Tables

**Table 1:** Elemental composition of GH4169.

Chemical Composition	Ni	Cr	Mo	Nb	Ti	Al
Percentage of elements	50.8%	17.74%	2.96%	4.93%	0.85%	0.68%

**Table 2:** The physical properties of GH4169.

Material Indicators	Tensile Strength	Yield Strength	Elongation	Shrinkage	Specific Gravity	Durometer
Performance Parameters	1280 MPa	1030 MPa	12%	15%	8.24 g/cm^3^	450 HBS

**Table 3:** Table of spray parameters.

Cutting Parameters: Cutting Speed *v* = 100 m/min, Feed Rate *f* = 0.2 mm/r, Cutting Depth *a_p_* = 0.25 mm		
Serial Number	Spray Pressure (MPa)	Spray Flow Rate (L/h)
1	0.1	2
2	0.2	2
3	0.4	2
4	0.2	3
5	0.2	3.5

**Table 4:** Relative error between experimental and simulated residual stress values.

Sample	Parameters	Residual Tensile Stress Value (MPa)			Residual Compressive Stress Value (MPa)		
		Measured	Simulated	Relative Error	Measured	Simulated	Relative Error
1	0.1 MPa	1108.46	1115.51	0.6%	−387.29	−393.74	1.6%
2	0.2 MPa	1049.68	1027.05	2.1%	−367.56	−351.01	4.5%
3	0.4 MPa	930.83	933.93	0.3%	−293.42	−302.01	2.9%
4	2 L/h	1049.68	1023.95	2.4%	−367.56	−346.11	5.8%
5	3 L/h	939.5	928.98	1.1%	−290.12	−284.30	2.0%
6	3.5 L/h	1124.5	1099.15	2.3%	−261.15	−267.81	2.6%

## Data Availability

Data are contained within the article.
